# Spiritual quality of life in family carers of patients with advanced cancer—a cross-sectional study

**DOI:** 10.1007/s00520-021-06080-5

**Published:** 2021-03-04

**Authors:** Ingebrigt Røen, Anne-Tove Brenne, Cinzia Brunelli, Hans Stifoss-Hanssen, Gunn Grande, Tora Skeidsvoll Solheim, Stein Kaasa, Anne Kari Knudsen

**Affiliations:** 1 Department of Clinical and Molecular Medicine, Faculty of Medicine and Health Sciences, Norwegian University of Science and Technology (NTNU), Trondheim, Norway; 2 St. Olavs Hospital, Trondheim University Hospital, 4. etg. Kunnskapssenteret vest, St. Olavs Hospital, 7006 Trondheim, Norway; 3 Chaplaincy, St. Olavs Hospital, Trondheim University Hospital, Trondheim, Norway; 4 Cancer Clinic, St. Olavs Hospital, Trondheim University Hospital, Trondheim, Norway; 5 Palliative Care, Pain Therapy and Rehabilitation Unit, Fondazione IRCCS Istituto Nazionale Tumori, Milano, Italy; 6 Center of diakonia and professional practice, VID Specialized University, Oslo, Norway; 7 Division of Nursing, Midwifery and Social Work, The University of Manchester, Manchester, England; 8 European Palliative Care Research Centre (PRC), Oslo University Hospital and University of Oslo, Oslo, Norway; 9 Institute of Clinical Medicine, Faculty of Medicine, University of Oslo, Oslo, Norway; 10 Department of Oncology, Oslo University Hospital, Oslo, Norway; 11 University of Oslo, Oslo, Norway

**Keywords:** Spiritual quality of life, Family caregivers, Advanced cancer, Palliative, Spiritual care, Quality of life

## Abstract

**Purpose:**

Caring affects carers’ psychological and physical health, mortality, and quality of life (QoL) negatively. Lower spiritual QoL is associated with anxiety and depression, but the spiritual dimension is rarely investigated in carers. The present study aimed to explore which patient- and carer-related characteristics were associated with spiritual QoL in carers of patients with advanced cancer.

**Methods:**

Secondary analyses were conducted using data from a prospective study investigating integration between oncology and palliative care. Adult patients with advanced cancer and their carers were included, and baseline data considering demographics, clinical characteristics, symptoms, social support, and religious meaning-making were registered. Spiritual QoL was measured using the Functional Assessment of Chronic Illness Therapy - Spiritual well-being (FACIT-Sp-12) questionnaire. Associations to spiritual QoL were explored by bivariate and multivariate regression models.

**Results:**

In total, 84 carers were included, median age was 62.5 years, 52 (62%) were female, and the average spiritual QoL score was 23.3. In bivariate analyses, higher education, social support, and lower patients’ symptom burden were significantly associated with higher spiritual QoL. The multivariate regression model (*n*=77) had an explained variance (*R*^2^) = 0.34 and showed a significant association for social support, higher education, having children < 18 years living at home, and patient’s age.

**Conclusion:**

The study indicates that spiritual QoL in carers were low and were negatively affected by several factors related to both carers and patients. However, there could be other important factors not yet described. Health care professionals should be aware of the known associated factors, as carers who hold these may need extra support.

## Background

Family caregivers (hereafter: carers) have been defined as follows: “Carers, who may or may not be family members, are lay people in a close supportive role who share in the illness experience of the patient and who undertake vital care work and emotion management” [1]. Improved treatments have extended the period of time carers spend caring for patients with advanced cancer, and the demands on carers have consequently increased considerably [2]. The burden of caring often exceeds carers’ coping abilities [3] and has been reported to negatively affect the carers’ psychological and physical health, mortality, social life, and quality of life (QoL) [4, 5].

**Fig. 1:**
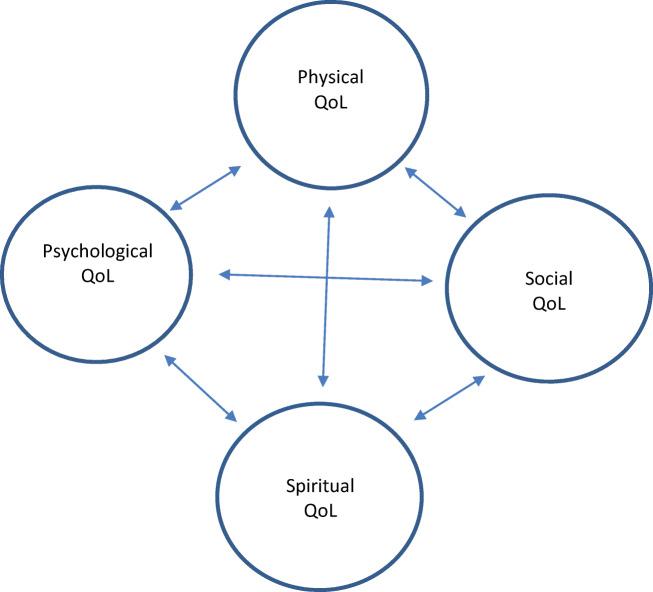
The four dimensions of quality of life (QoL). Changes in one QoL dimension may influence the other dimensions

The World Health Organization (WHO) includes spirituality in its four-dimensional palliative care definition (physical, psychological, social, and spiritual dimension) [6]. Changes in one dimension may influence one or more of the other dimensions (Fig. 1). Puchalski et al. stated that chaplains, or other spiritual experts, should be integrated in the health care team and recognized and referred to as the spiritual experts. Furthermore, they stated that all professions must share the responsibility for assessment and treatment of spiritual suffering [7].

One challenge of investigating the impact of spirituality in palliative care is the lack of clarity of its definitions [8]. Earlier, spirituality was understood mainly as religiosity. Today, religiosity is understood more as a potential sub-category of a person’s spirituality [9]. Despite this, research on spirituality in palliative care has often concentrated on religiosity, mainly Christian. In the present study, we defined spiritual QoL without a religious component, in line with the European Association for Palliative Care (EAPC) Taskforce on spiritual care definition: “Spirituality is the dynamic dimension of human life that relates to the way persons (individual and community) experience, express and/or seek meaning, purpose and transcendence, and the way they connect to the moment, to self, to others, to nature, to the significant and/or the sacred” [10].

However, even though focus on spirituality has been recommended [11, 12], it is still rarely included in palliative care research [9, 12]. Few studies have explored factors that impact on the spiritual QoL of carers of patients with advanced cancer, despite the existential threat they are exposed to. In a study including 41 carers of patients with advanced cancer, the 23 (58%) reporting having spiritual pain**,** had poorer scores in anxiety, depression, and more dysfunctional coping strategies than those not reporting spiritual pain [13]. Another study including 199 carers of cancer patients reported that spiritual QoL was associated with bodily pain, mental, and social QoL [14]. Furthermore, studies have shown an association between spiritual Qol in carers and their social support [15, 16], levels of anxiety [16, 17], levels of depression [17], and mental health [18, 19].

It is imperative to improve the QoL of the growing populations of carers of patients with advanced cancer, but there are neither resources nor need to offer all carers the same level of care. The overall aim of the present study was thus to identify carers of patients with advanced cancer who may need extra support. The following research question was addressed: Which patient- and carer-related characteristics were associated with spiritual QoL of carers?

## Methods

### Participants and study design

Secondary analyses were conducted using data from a prospective controlled intervention trial investigating integration between oncology and palliative care, “the Orkdal Model [20] (ClinicalTrials.gov Identifier: NCT02170168). The “Orkdal Model” aimed to investigate early integration between oncology and palliative care across specialist and community care in Mid-Norway. In the Orkdal study, adult cancer patients from 22 municipalities, not receiving curative treatment, were recruited from home care, nursing homes, and hospital. All adult patients were asked for written, informed consent to approach their primary carers aged 18 years or more for participation in the study. Thereafter, all eligible carers were asked to give written, informed consent to participate. All carers filling in baseline assessments were eligible for analyses in the present study.

### Data collection and assessments

Patients reported gender, marital status, and patient reported outcome measures (PROMs). Symptom intensities were assessed according to the EAPC Basic Dataset [21]. Symptom burden was measured as the number of symptoms the patients scored > 5 on a 0–10 numerical rating scale NRS (0–10) where low scores indicate low symptom intensity. Health care professionals (HCPs) reported patients’ age, primary cancer disease, stage, comorbidity [21], expected survival, Karnofsky Performance Status (KPS) [22], and treatment setting (home, nursing home, hospital). Carers reported year of birth, gender, relation to the patient, having children <18 years living at home, if they were living with the patient, highest education completed, employment, caring for others in addition to the patient, religious affiliation, and use of voluntary services.

Spiritual QoL was measured using the Functional Assessment of Chronic Illness Therapy - Spiritual well-being (FACIT-Sp-12) questionnaire [23]. FACIT-Sp-12 is a 12-item self-report questionnaire that was developed and validated in 1617 chronically ill patients, the majority (83.1%) having a cancer diagnosis. The first eight items forming the meaning/peace subscale were applied as outcome in the present study. The four items’ faith subscale was not used, since religious QoL was not part of our study. Each item was rated on a 5-point Likert scale ranging from not at all to very much. The total score of the meaning/peace scale ranged from 0 to 32; higher scores indicated higher spiritual QoL. Mean score in the validation study was 25.2 [23].

Carers’ perceived social support from family, friends, and other social network was measured using an adapted version of the last four of the 20 items’ MacAdam’s Initial Assessment of Suffering Scale (IAS) **[**24]. IAS was developed to assess suffering in seriously ill patients and was validated in patients with advanced cancer [24]. Each item was scored on a 4-point Likert-scale ranging from “not at all” to “very much,” range 4–16; higher scores indicated better social support. Reference scores from validation studies for the items used were not available.

How important religious meaning-making was to the carers was measured using three items from “Vertical self-transcendence - explicit religiosity” subscale from The Sources of Meaning and Meaning in Life Questionnaire (SoMe) [25–27]. A 6-point Likert-scale was used, ranging from “not at all true” to “completely true,” range 0–15. SoMe has been validated in a representative population in Germany [26] and Norway [25]. In Norway, the mean score was 4.53 for the three items used in our study: “religion plays an important role in my life,” “prayer is important to me,” and “my religion gives me strength” [25].

## Statistical analysis

Basic descriptive statistics (mean range and standard deviation) as well as frequency distributions were used to summarize patient and caregiver related data. The main study aim was addressed with bivariate and multivariate linear regression models in which carers’ spiritual QoL was handled as dependent variable (main outcome of interest). Based upon existing literature, the following factors were used as independent variables in regression models: (a) patient-related factors: age, gender, KPS, comorbidity, time since diagnosis, expected survival > 1year, symptom burden; (b) carer-related factors: children <18 years living at home, living with patient, highest education completed, social support, and religious meaning-making. Normal Q-Q plot, scatterplot of the residuals, and Variance Inflation Factor (VIF) values for multicollinearity among independent variables were examined as regression model diagnostics. Linear regression results were reported in terms of estimated regression coefficients beta, corresponding 95% confidence interval (95% CIs) and overall *p* values. The conventional two-sided 5% level was chosen as the threshold of statistical significance. Adjusted *R*^2^ was used as indicator of the amount of variance in the outcome explained by independent variables examined. Analyses were carried out using IBM SPSS Statistics 25 (Statistical Product and Service Solutions) and STATA Statistical Software (Release 16; StataCorp LP, College Station, TX).

## Results

**Fig. 2:**
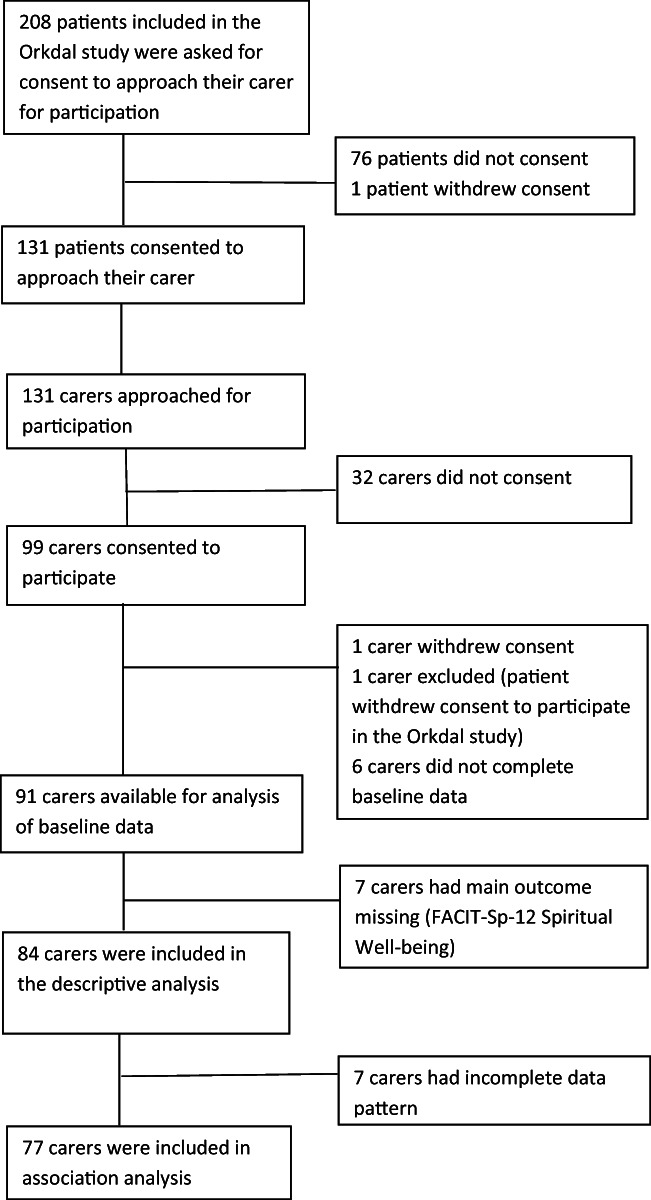
Attrition of patients and carers

Patients and carers were included from Nov. 2014 to December 2017. Among 208 patients included in the study and available for data analysis, 131 consented to their carers being approached for participation. Not wanting to overload their carer was the patients’ main reason for not consenting. Out of 131 carers approached, 99 consented to participate and completed the baseline assessments. Feeling overloaded was the carers’ main reason to decline participation. Seven carers had incomplete assessment of FACIT; thus, 84 carers were included in the descriptive analysis and in the bivariate regression analysis. Due to missing data in one or more of the variables included in the multivariate analysis, another seven carers were excluded, resulting in a final analysis sample of 77 carers included in the multivariate analysis (Fig. 2).

The patients’ and carers’ characteristics were described in Tables 1 and 2. Mean age of the patients was 69.7 years and 46 (55%) were men. Carers’ (*n*=84) mean age was 62.5 years, 52 (62 %) were female, and 63 (75 %) were patients’ partners. Ten (12 %) had children < 18 years living at home.

**Table 1:** Patient characteristics (*N*=84)

Patient characteristics	*N* (%)	Mean (range)	SD
Age		69.7 (42–92)	9.9
Gender			
Male	46 (54.8%)		
Female	38 (45.2%)		
Cancer diagnosis			
Gastro-intestinal	31 (36.9%)		
Genito urinary	19 (22.6%)		
Breast	13 (15.5%)		
Lung	8 (9.5%)		
Other	13 (15.5%)		
Time since diagnosis		3.1 months (0.1–15.4)	
Marital status			
Married/cohabitant	69 (82.1%)		
Widow/widower	10 (11.9%)		
Divorced/separated	3 (3,6%)		
Single	2 (2.4%)		
Highest education*			
Primary school	27 (32.1%)		
Secondary school	34 (40.5%)		
College/university	20 (23.8%)		
Stage of cancer			
Metastatic	71 (84.5%)		
Local advanced	11 (13.1%)		
Local or complete remission	2 (2.4%)		
Cancer treatment ongoing*			
Yes	76 (90.5%)		
No	5 (6%)		
Treatment setting*			
Outpatient	55 (65.5%)		
Inpatient	18 (21.4%)		
Nursing home	2 (2.4%)		
Karnofsky performance status		82.2 (20–100)	
Symptom burden			
At least one symptom > 5			
Yes	44 (53.7)		
No	38 (46.3)		
Symptom intensity (NRS 0–10)		1.9 (0–6)	1.5
Number of symptoms NRS>5		1.9 (0–9)	2.3

*Percentage does not add up to 100 due to some missing registrations

**Table 2:** Carer characteristics (*N*=84)

Carer characteristics	*N* (%)	Mean (range)	SD
Age		62.5 (35–85.6)	11.7
Gender			
Female	52 (61.9%)		
Male	32 (38.1%)		
Relation to patient			
Partner	63 (75%)		
Parent	9 (10.7%)		
Daughter/son	7 (8.3%)		
Sibling	3 (3.6%)		
Parent in law	1 (1.2%)		
Other	1 (1.2%)		
Children <18y living at home	10 (11.9%)		
Living with the patient^a^			
Yes	58 (69.1%)		
No	23 (27.4%)		
Highest education			
Primary school	12 (14.3%)		
Secondary school	39 (46.4%)		
College/university	32 (38.1%)		
Missing	1 (1.2%)		
Employment^a^			
Pension	35 (41.7%)		
Employed	32 (38.1%)		
Disabled	4 (4.8%)		
Other	7 (8.3%)		
Carer for others in addition to the patient	9 (10.7%)		
Church member	79 (94%)		
Use of voluntary services			
Yes^b^	4 (4.8%)		
No	80 (95.2%)		
Social support		11.32(6–16)	2.43
Religious meaning- making		4.11(0–15)	4.76
Spiritual QoL		23.34 (13–32)	4.48

^a^Percentage does not add up to 100 due to some registrations missing; ^b^Several options possible

Patients’ mean symptom intensity (NRS 0–10) was 1.9, 44 of 82 (46 %) had at least one symptom with NRS score > 5, and the mean number of symptoms > 5 was 1.9.

Spiritual QoL, measured as average meaning/peace scores, were reported by carers as 23.3 (range 13–32) (Table 2 and 3), which is lower than in the validation study (25.2) [23]. Social support was 11.3 (range 6–16) (Tables 2 and 3). The importance of religion as a source of meaning-making for the carers had a mean score of 4.11 (range 0–15) (Tables 2 and 3), which is lower than 4.53 reported among a representative population in a Norwegian validation study [25].

**Table 3:** Carers’ spiritual quality of life, social support, and religiosity

Items	*N*	Mean (range)	SD
The Functional Assessment of Chronic Illness Spiritual Well-being Scale (FACIT-Sp)			
I feel peaceful	75	2.16	0.89
I have a reason for living	76	3.61	0.61
My life has been productive	77	3.14	0.87
I have trouble feeling peace of mind^a^	74	1.03	1.01
I feel a sense of purpose in my life	75	3.24	0.77
I am able to reach down deep into myself for comfort	73	2.51	0.92
I feel a sense of harmony within myself	71	2.30	0.76
My life lacks meaning and purpose^a^	73	0.49	0.93
Total		23.3 (13–32)	4.48
Scored on a 5-point scale ranging from 0 (not at all) to 4 (very much) ^a^Item with reversed score			
MacAdam’s Initial Assessment of Suffering Scale (IAS) is a 20-item scale			
The support I have had from my family and friends has been	81	3.23	0.83
I have felt needed by my family and friends	81	2.75	0.85
I have been able to share how I am feeling now with another person	80	2.75	0.95
Contacts outside my family, e.g., church, work, club etc. have been	81	2.90	0.72
Total		11.32 (6–16)	4.48
Scored on a 4-point Likert-scale ranging from not at all (1) to very much (4)			
The Sources of Meaning and Meaning in Life Questionnaire (SoMe), the “Vertical self-transcendence - explicit religiosity” subscale			
Religion is an important part of my life	78	1.58	1.59
It is important for me to pray	78	1.14	1.63
I draw strength from my faith	79	1.33	1.66
Total		4.11 (0–15)	4.76

Scored on a 6-point Likert-scale, ranging from “not at all true” to “completely true”

In bivariate analyses (Table 4), higher education, higher social support, and lower patient symptom burden were significantly associated with higher spiritual QoL in carers. In multivariate analyses (Table 4), higher education, higher social support, having children living at home, and older patient age showed significant association with higher spiritual QoL. Hence, two of the three significant associations from the bivariate analyses were confirmed in the multivariate model: higher education, and social support. The association between lower patient symptom burden and carers’ higher spiritual QoL was still high in the multivariate analysis, but not significant (*p* = 0.068). Two additional significant associations with better carers’ spiritual QoL were found: older age of patients, and having children < 18 years living at home. None of the regression diagnostics indicated violation of linear regression assumption and the explained variance (adjusted *R*^2^) was 0.34.

**Table 4:** Bivariate and multivariate analyses of carers’ spiritual quality of life

	Beta	CI	*p*
Bivariate analyses (*N*=84)			
Carer variables			
Gender			
Female	0.10	−1.91, 2.11	0.921
Children <18y living at home			
Yes	2.68	−0.28, 5.64	0.075
Living with the patient			
Yes	−0.41	−2.54, 1.73	0.705
*Highest education*			
High school	2.07	−0.78, 4.93	0.152
*College/university*	*3.71*	*0.79, 6.63*	*0.013*
*Social support*	*0.54*	*0.15, 0.93*	*0.007*
Religious meaning-making	0.10	−0.11, 0.31	0.347
Patient variables			
Time from diagnosis	0.04	.0.22, 0.29	0.778
Patient age	0.08	−0.02, 0.18	0.101
Karnofsky	0.02	0.05, 0.09	0.574
*Symptom burden*	*−0.50*	*−0.91, −0.08*	*0.020*
Comorbidity	0.19	−2.30, 2.67	0.882
Expected survival			
>1 year	0.57	−1.76, 2.90	0.627
Multivariate analyses (*N*=77)			
Carer variables			
Gender			
Female	−1.21	−3.32, 0.90	0.258
*Children <18y living at home*			
*Yes*	*3.79*	*0.84, 6.74*	*0.013*
Living with the patient			
Yes	1.62	−0.88, 4.12	0.200
*Highest education*			
*High school*	*3.30*	*0.14, 6.47*	*0.041*
*College/university*	*4.32*	*1.08, 7.55*	*0.010*
*Social support*	*0.42*	*0.002, 0.85*	*0.049*
Religious meaning-making	0.07	−0.14, 0.28	0.493
Patient variables			
Time from diagnosis	0.16	−0.10, 0.41	0.236
*Patient age*	*0.13*	*0.016, 0.25*	*0.026*
Karnofsky	0.019	−0.06, 0.10	0.628
Symptom burden	−0.41	−0.85, 0.03	0.068
Comorbidity	−0.93	−3.74, 1.87	0.509
Expected survival			
>1 year	−0.38	−2.82, 2.07	0.760

Significant values are in italics

Religious meaning-making was not significantly associated with levels of experienced meaning and peace neither in bivariate nor multivariate analysis.

## Discussion

Aiming to improve palliative care services, baseline data from the Orkdal Model trial were investigated to improve the identification of carers in need of extra support. Carers’ education level, social support, having children living at home, and patients’ age were significantly associated with spiritual QoL of carers. Patients’ symptom burden and age were significantly associated with carers’ spiritual QoL in bivariate analyses, but not in multivariate analyses (*p*=0.068 and 0.101 respectively). Variation of results in bivariate and multivariate analyses may be due to a relatively low strength due to small sample size.

Data from the present study can be used to identify current carers who may need extra support to improve their QoL. However, the explained variance was 34%, suggesting that also other factors than the ones investigated in the present study were of importance.

Overall, the carers in the present study reported a low mean spiritual QoL compared with other studies. Firstly, the carers’ spiritual QoL was lower than the reference value scores among 8864 cancer survivors (23.3 vs 25.7) [28]. Furthermore, the score in the present study was lower than the corresponding 25.2 reported by 1617 patients investigated in the FACIT-Sp validation study [23]. Carers of patients with advanced cancer having poorer spiritual QoL scores than patients with advanced cancer should be a reminder for health care professionals (HCPs) of carers’ burdens and their need for support. Another study showed that carers of patients with advanced cancer, describing themselves as having spiritual pain, had a mean score of 24.0 (20–25), while carers with no spiritual pain reported 28.0 (24–30) [13]. Compared to that study, the spiritual QoL level of the majority of carers in our study could imply that they suffered from spiritual pain.

An association between education level and spiritual QoL has to our knowledge not previously been reported in carers of patients with advanced cancer. However, it is well known that higher education level is associated with better physical and mental health in the general population [29]. Carers with higher education level may profit more from information given by HCPs and are probably more informed about available support and how to access it. A study among carers of patients with advanced cancer reported that lower education was associated with more emotional distress [30]. A recent review reported that psychosocial and/or educational interventions have shown to give a significant positive effect on carers’ QoL, psychological distress, and coping [31], and HCPs should thus do an extra effort to support carers with lower education level, including providing additional information and education.

Carers may need support since caring for patients with advanced cancer is demanding, affecting their QoL, health, and social life negatively [4, 5]. It has been suggested that interventions including social support should be developed to buffer against low spiritual QoL [17]. In the present study, a positive association was found between higher social support and higher spiritual QoL. This has also been reported among carers of patients with newly diagnosed cancer [15]. Social support may include both emotional and practical support. Whereas the bulk of social support is provided by family, friends, and neighbors [32], HCPs may also have significant contributions, including their own relationship to the carers [33, 34]. Relationships may be regarded as spiritual support and meaning in itself [35]. Providing extra support to carers with lower social support could imply spending extra time, building a trustful relationship, and involvement of the interdisciplinary team. HCPs should additionally consider activating other parts of the carers’ network, e.g., family members, friends, neighbors, colleagues, and faith community. On an organizational level, systematic recruitment, training, and use of volunteers to support carers should be considered [36]. The most important social support for many carers is the relationship with the patients themselves, and HCPs should facilitate the interaction and communication between carer and patient [33]. Family conflict may hamper the family’s support to the carer. Hence, HCPs may consider to contribute positively, e.g., by organizing a family meeting, and/or by referral to a social worker or a chaplain.

The positive association between having children < 18 years living at home and higher spiritual QoL was another finding that underlines the significance of social support and relationships. According to the European Association for Palliative Care (EAPC) definition of spirituality, we “experience, express, and/or seek meaning, purpose and transcendence” in our relationships [10]. Caring for children is for most parents undoubtedly associated with purpose and meaning in life. Furthermore, children divert thoughts, fill daily life with activities and fellowship, and represent in themselves hope for the future. Also, carers know that the patient will live on in the children’s memories and in the carers’ future communication with them. Contrary to this, carers without children living at home know they face a near future living alone. Given the reported importance of social support for spiritual QoL in this study, and of trustful relationships for carers’ resilience in another study [33], helping maintain carers’ close relations to their children should be prioritized. HCPs should regard carers’ children as a resource for carers, support the children, and help carers communicate with their children [37]. There might be differences as to whether children improve or reduce the burden for carers dependent on children age, but the sample size did not allow for a comparison of age groups.

To our knowledge, an association between patients’ age and carers’ spiritual Qol has not been reported in other studies in advanced cancer. Younger age of patients approaching death might often be more distressing for carers than the imminent death of older patients, and may be experienced as more meaningless, unfair, and as being against the natural life course. Older people dying may be seen as more acceptable, since they have lived a long life. Carers are normally more prepared when older people die, and might more easily reconcile with it.

The present study could not confirm that patients’ symptom burden was associated with spiritual QoL in multivariate analysis. Differently, among cancer survivors and their carers, Kim et al. found a significant association between survivors’ physical health and carers’ spiritual QoL [18]. The divergent findings in the two populations might be due partly to the fact that carers in our study were aware that the patient would die, independently of symptom burden.

The relationship between religious meaning-making and spiritual QoL has to our knowledge not earlier been investigated among carers of patients with advanced cancer. Such a correlation could have been expected in our study, since 94% were members of the Lutheran church of Norway (71% nationally in 2018) [38], and a survey among church members in the actual diocese in 2019 found that 82% held open the possibility of God’s existence, 49% prayed, and 87% attended church at least once the previous year [39]. However, the mean score of religious meaning-making in our study (4.11) was lower than in the validation study in a Norwegian normal population (4.53) [25], and our study did not find a correlation between religious meaning-making and spiritual QoL. Among palliative care patients, a positive association between religious coping and QoL has likewise not been found [9]. From the present data, it was not possible to explore if religion would have more impact in the terminal phase.

Even though it has been stated that all professions must share the responsibility for treatment of spiritual suffering [7], only 45% of Australian oncologists and oncology trainees reported to be able to meet spiritual needs. Lack of time, education and understanding of spirituality, and spiritual care were perceived as barriers for providing spiritual care [40]. Likewise, nurse- and physician-reported lack of education was identified as the primary barrier to spiritual care provision in the terminal phase of cancer [41]. A new review of recent European literature on spiritual care in palliative care concluded that implementation of spiritual care required spiritual competency, and recommended education of health care professionals, hereunder self-reflection. Furthermore, it was regarded paramount to increase the visibility of spiritual care, and that implementing spiritual counselors as part of the palliative care teams was one of the options to achieve this [42]. Spiritual QoL should be integrated as a standard component in QoL research in palliative care and in clinical palliative care [12]. Being experts on spirituality, spiritual care, and existential conversations [7], the (religious and non-religious) representatives of the chaplaincy staffs should engage in spiritual QoL research, and educate other health care professionals in spiritual care. Chaplaincy-led, multidisciplinary training in spiritual care has been found to be feasible in hospitals [43].

The present study was performed in two similar rural parts of Mid-Norway where no carers had cultural background other than Norwegian, reducing the generalizability of the results. Firstly, relatively few patients and carers were included in this study, which challenge the generalizability of the findings. Additionally, an increased sample size would have allowed more statistical power to the analyses of associations. Furthermore, it would have been of value to investigate prospective data rather than cross-sectional. There were few validated assessment tools evaluating spiritual QoL in carers of patients with advanced cancer available when the study was initiated. Originally, FACIT-Sp-12 was not developed for carers. It was still chosen for our study since it is well validated in an advanced cancer population, and has previously been used in several carer studies [15–19]. In a review of 35 instruments measuring spirituality in clinical research, this questionnaire was found to be among the two best instruments for assessing spiritual QoL [44]. Due to slightly altered wording and scoring of the four items adapted from MacAdam’s Initial Assessment of Suffering Scale (IAS), reference values from validation studies could not be used. However, the items were implemented since we regarded them useful for exploring how social support was associated to spiritual QoL. The definition of spiritual QoL used in the present study is applicable in both religious and non-religious populations, thus representing a strength. Other strengths were that the study explored a field of little previous research and described factors associated with the spiritual QoL in carers of patients with advanced cancer not reported before.

## Conclusions

The present results indicate that spiritual QoL is likely to be lower in carers with low education level and low social support, not having children living at home, and also for carers of younger patients. Health care professionals (HCPs) should be aware that these carer groups may need extra support. HCPs should provide spiritual care by building a trustful relationship, spending extra time with them, providing extra information and education, and involving others in the interdisciplinary team, and community care at an early stage. Furthermore, family, friends, and relevant institutions such as faith organizations should be mobilized. To progress palliative care, we recommend spiritual care education to all palliative team members, and to initiate clinical trials systematizing psycho-social and educational interventions aiming to improve carers’ four-dimensional QoL.

## Declarations

### Code availability

Analyses were carried out using IBM SPSS Statistics 25 (Statistical Product and Service Solutions), and Stata 16.0 for Windows (Stata Statistical Software; Stata Corporation, College Station, Texas).
